# Adenosine 2 receptor regulates autophagy and apoptosis to alleviate ischemia reperfusion injury in type 2 diabetes via IRE-1 signaling

**DOI:** 10.1186/s12872-023-03116-y

**Published:** 2023-03-24

**Authors:** Mohamed Bassirou Yacouba Moukeila, Erick Thokerunga, Feng He, Christian Cedric Bongolo, Yun Xia, Fuyu Wang, Adamou Foumakoye Gado, Hama Mamoudou, Shahzad Khan, Bonkano Ousseina, Hadjara Abdoulkarim Ousmane, Drissa Diarra, Jianjuan Ke, Zongze Zhang, Yanlin Wang

**Affiliations:** 1 Department of Anesthesiology, Zhongnan Hospital of Wuhan University, Wuhan, 430071 Hubei China; 2 Program and Department of Clinical Laboratory Medicine, Center for Gene Diagnosis, Zhongnan Hospital of Wuhan University, Wuhan, 430071 Hubei China; 3 Department of Anesthesia and Intensive Care, Hôpital Général de Référence Niamey, Niamey, Niger; 4 Department of Anesthesia and Intensive Care, National Hospital of Niamey, Niamey, Niger; 5 Department of Pathology, Zhongnan Hospital of Wuhan University, Wuhan, 430071 Hubei China; 6 Department of Cardiovascular and Internal Medicine, Niamey Amirou Boubacar Diallo National Hospital, Abdou Moumouni University, Niamey, Niger; 7 Department of Oral and Maxillofacial Surgery, School and Hospital of Stomatology, Wuhan University, Wuhan, China

**Keywords:** Adenosine2 receptor, Myocardium, Ischemia, Reperfusion, Endoplasmic reticulum stress

## Abstract

**Purpose:**

This study aimed to determine the effect and mechanism of action of adenosine 2 receptor (A2R) activation on myocardial ischemia reperfusion injury (MIRI) under diabetic conditions.

**Methods:**

MIRI type 2 diabetic rats and H9C2 cardiomyocytes were treated with A2R agonist and then subjected to hypoxia for 6 h and reoxygenation for 18 h. Myocardial damage, and infarct size were determined by cardiac ultrasound. Indicators of cardiomyocyte injury, creatine kinase-MB and cardiac troponin I were detected by Enzyme Linked Immunosorbent Assay. Endoplasmic reticulum stress (ERS) was determined through measuring the expression levels of ERS related genes GRP78, p-IRE1/IRE1, and p-JNKJNK. The mechanism of A2R cardio protection in MIRI through regulating ERS induced autophagy was determined by investigating the ER resident protein IRE-1. The ER-stress inducer Tunicamycin, and the IRE-1 inhibitor STF in combination with the A2R agonist NECA were used, and the cellular responses were assessed through autophagy proteins expression Beclin-1, p62, LC3 and apoptosis.

**Results:**

NECA improved left ventricular function post MIRI, limited myocardial infarct size, reduced myocardial damage, decreased cardiomyocytes apoptosis, and attenuated ERS induced autophagy through regulating the IRE-XBP1s-CHOP pathway. These actions resulted into overall protection of the myocardium against MIRI.

**Conclusion:**

In summary, A2R activation by NECA prior to ischemia attenuates apoptosis, reduces ERS induced autophagy and restores left ventricular function. This protective effect occurs through regulating the IRE1-XBPs-CHOP related mechanisms. NECA is thus a potential target for the treatment of MIRI in patient with type 2 diabetes.

**Supplementary Information:**

The online version contains supplementary material available at 10.1186/s12872-023-03116-y.

## Introduction

Myocardial infarction (MI) is an ischemic heart condition whose risk remains very high among type 2 diabetic (T2DM) patients [1, 2]. Indeed, T2DM patients are twice as likely to suffer ischemic heart disease, and have a significantly raised post MI mortality rate compared to non-diabetics [3, 4]. First line treatment for acute myocardial ischemia to save the ischemic myocardium is reperfusion. However, this treatment is often associated with tissue injury in a condition called “myocardial ischemia reperfusion injury” (MIRI). Various factors are responsible for MIRI including cellular autophagy, which decreases the number of cardiomyocytes needed to repair cardiac systolic function [5]. Moreover, T2DM has been shown to exacerbate endoplasmic reticulum stress mediated autophagy in the pancreas [6]. Therefore, inhibition of autophagy could be a way of alleviating MIRI.

During MI, the extracellular concentration of the purine nucleoside molecule, Adenosine, has been found to dramatically increase, indicating a possible function in MI [7]. Studies have shown that binding of adenosine to its receptors (A1, A2a, A2b and A3); that are extensively expressed on the mammalian myocardium, has beneficial effects on MIRI [8]. Adenosine 2a receptor in particular is cardioprotective during reperfusion and this cardio protection is associated with downregulated autophagy [9]. A recent study demonstrated that activation of A2R in diabetic rats protects against MIRI by preventing apoptosis [10]. Whether the endoplasmic reticulum stress induced autophagy is involved is not yet clear.

Endoplasmic reticulum stress (ERS) activates a process called autophagy, where nutrients are recycled through lysosomal degradation of unfolded or misfolded proteins and worn out cellular organelles [11]. Under extreme stress such as those induced by MIRI, autophagy is activated to encourage cellular survival and energy conservation [12]. However, autophagy is a double edged sword; enhanced autophagy following MIRI can lead to significant reduction of viable cardiomyocytes which impairs recovery of ventricular functions and exacerbate the condition. In normal cells, activation of A2R promotes cardio-protection through regulating autophagy [9]. Whether the ER is involved has not been elucidated. Previous studies have demonstrated that the endoplasmic reticulum membrane protein, IRE-1 regulates autophagy in various cells in response to ER- stress [13–15]. In this study, we examined whether A2R activation regulates ERS induced autophagy in diabetic rats to offer cardio protection during MIRI, and the mechanism involved.

## Materials and methods

### Animals

Fifty Sprague Dawley (SD) adult rats (250–280 g) were offered by Hubei Experimental Animal Research Center for this experiment (License No.: SCXK (E) 2015-0018). All animal experiments were approved by the Animal Experiment Committee of Wuhan University (Ethical approval number: WP2020-01108), and strictly conducted in compliance with the study protocol, the ARRIVE guidelines, and the Guide for the Care and Use of Laboratory animals (No. 85-23, revised 1996) published by National Institutes of Health (NIH).

### H9C2 myocardial cells

The rat embryonic cells H9C2 cells were provided by Procell (Wuhan, China) and maintained in Dulbecco’s Modified Eagle Medium (DMEM, Thermofischer scientific, USA) supplemented with 10% fetal bovine serum (FBS, Life technologies, Carlsbad, Ca, USA) and 1% Streptomycin-Penicillin antibiotics (FBS, Life technologies, Carlsbad, Ca, USA). Cells were cultured at 37 °C in 5% CO_2_.

### Establishment of ischemia reperfusion injury model in vitro

After 72 h of culture, the neonatal rat cardiomyocytes (NRCM) were subjected to hypoxia reperfusion (H/R) to induce ischemia reperfusion injury. The cells were cultured for 6 h in a glucose-free, serum-free medium in the presence of 95% N_2_ and 5% CO_2_. Cells were then re-oxygenated for 18 h in fresh DMEM/F12 supplemented with 10% FBS, 1% penicillin–streptomycin, 1% BrdU an incubated at 37 °C and 5% CO2 [29].

### Measurement of autophagic flux

One day prior to ischemia reperfusion (I/R), the H9C2 cells were transfected with RFP-GFP-LC3 and LAMP-2 for 24 h to observe co-localization. Immediately after reoxygenation, cells were fixed with 4% paraformaldehyde, and then stained with DAPI. Confocal microscope (LEICA TCS SP8, Germany) was used to observe the fluorescence signal under multiple visions; yellow spots representing autophagosomes, and red spots representing autolysosomes. Images were analyzed using image J software.

### Measurement of endoplasmic reticulum stress

Cultured in a six plate well were removed, culture medium discarded and the cells washed 3 times with PBS for 5 min each. Cells were then fixed for 20 min with 4% paraformaldehyde, and then washed again 3 times with PBS for 5 min each. A histochemical pen was used to draw a circle to prevent the incubation solution from flowing away in the following process. Cells were then incubated overnight at 4 °C in primary antibodies (GRP8, dilution: 1: 1000; p-IRE1, dilution: 1: 500; XBP1, dilution: 1: 500) diluted with 5% BSA. The following day, they were washed 3 times with PBS for 5 min each and re-incubated in the dark in corresponding secondary antibodies (HRP-Goat anti Rabbit, and HRP-Goat anti mouse, dilutions: 1: 10,000 each) at 37 °C for 40 min, later washed with PBS and then stained with DAPI, incubated in the dark for 20 min at room temperature. They were washed with PBS and the film sealed with an anti-fluorescence quenching solution, then observed under the microscope.

### Detection of cell survival rate by CCK-8

Cells were cultured for 16 h at 9.5 × 10^5^/cm^2^ density in a 48-well plate, 10% CCK-8 solution was then added to the culture medium and further incubated for 2 h. A cell free control group was created and CCK-8 solution added as well. Absorbance was then measured at 450 nm (PerkinElmer EnSpire Microplate Reader, USA) and the optical density (OD) determined to calculate cell viability.

### RNA extraction, reverse transcription and qRT-PCR

Total RNA was extracted from the cardiomyocytes using TRIpure Total RNA Extraction kit (Cat: EP013) form ELK Biotechnology Co. Ltd., China according to the manufacturer’s instructions. Reverse transcription to make cDNA was conducted using M-MLV Reverse Transcriptase kit (Cat: EQ002) also from ELK Biotechnology Co. Ltd., China. Quantitative real-time PCR was performed using StepOne™ Real-Time PCR system (Life Technologies, CA, USA) using QuFast SYBR Green PCR Master Mix (Cat: EQ001, ELK Biotechnology Co.Ltd., China. A 10-μl total reaction volume was used, and the reaction were as follows: 95 °C for 1 min; 40 cycles of 95 for 15 s, 58 °C for 20 s and 72 °C for 45 s, melting curve 60 °C → 95 °C, with 1 °C temperature increase every 20 s. Beta-actin was used as the endogenous control for data normalization and the double delta method used to calculate gene expression. Ct values used for calculations were averages of three independent repeats. All primer information is provided in Additional file 1: Table S1.

### Establishment of diabetic rats’ model

To establish the diabetic model, SD rats were fed on a high fat diet containing 60% fats, 20% carbohydrates and 20% proteins for 30 days. After the 30 days, they were allowed to fast for 12 h, weighed and then injected with l% Streptozotocin citrate buffer at a 45 mg/kg dose. They continued to be fed on the high fat diet, and after 72 h, blood glucose from the tail veins were measured. The glucose levels were all greater than 16.7 mmol/L, indicating successful development of type 2 diabetes mellitus. The rats continued to be fed on high fat diet to maintain chronic type II diabetes (Additional file 2, Additional file 3, Additional file 4, Additional file 5 and Additional file 6).

### Myocardial ischemia-reperfusion injury model

To develop myocardial ischemia reperfusion models, each rat was weighed, and anesthetized by injection with 1% sodium pentobarbital intraperitoneally at 50 mg/Kg. A cut was then made along the midline of the neck, muscles and tissues separated, the trachea exposed and an inverted T-shaped incision made along the cartilage ring of the trachea to intubate it. Parameters of the ventilator were set as: VT = 30–50ML/; frequency = 50–70 times/min; and breathing ratio = 1:1. The left thorax was opened to expose the heart. A 5–0 suture was used to ligate the left anterior descending coronary artery (LAD) at about 2 mm under the junction between the pulmonary artery cone and the left atrial appendage. To ensure reversible LAD occlusion, an openable knot was created by passing a ploythene tube through it just before it was tightened. In the sham group, no ligation was conducted. Ischemia was confirmed when transient drop in blood pressure was noticed, and the surface of the myocardium turned blue, indicating cyanosis. Reperfusion recovery was demonstrated by the rapid disappearance of cyanosis and hyperemic responses in the epicardium. Occlusion was conducted for 30 min and reperfusion 120 min then the rats euthanized by anesthesia overdose.

### Hemodynamic measurements

Following anesthesia, while in the supine position, a puncture was made in the right internal carotid artery and an electrocardiogram connected. (BL-420, Taimeng Informatization Biological Signal Acquisition and Analysis System, China). 100U/Kg heparin anticoagulant was then injected and a catheter inserted into the right internal carotid artery to monitor the arterial blood pressure of the rat.

### Echocardiography

The GE Vivid 7 (GE Health Medical, USA), was used to conduct transthoracic echocardiography. It was fitted with an 11 MHz image transducer to generate images. The ultrasound personnel was blinded to the study. Data collected were on: structure of the left ventricle, systolic ventricular septal thickness (IVSs), diastolic ventricular septal thickness (IVSd), left ventricular end-systolic diameter (LVIDs), left ventricular end-diastolic diameter (LVIDd), left ventricular diastolic posterior wall thickness (LVPWd), and left posterior wall thickness (LVPWs). These were all collected by M-mode ultrasound, while left ventricular end-systolic volume (LVESV), Left ventricular end-diastolic volume (LVEDV), left ventricular ejection fraction (LVEF), stroke volume (SV) and left ventricular fractional shortening (FS) were calculated automatically using alogarithms in the computer.

### Measurement of myocardial infarction area (Evans blue staining)

Once reperfusion was complete, the reversible LAD occlusion was fully ligated. Large amounts of pentobarbital sodium was administered via the tail vain to euthanize the rats, while Evans blue dye was injected using the femoral vein. The hearts were removed, washed with phosphate buffered saline 3 times and then quickly frozen for 10 min at − 80 °C. The ventricular part was then sliced from the top into small equal parts. They were then incubated in 1% triphenyl tetrazolium chloride (TTC) for 15 min at 37 °C and allowed to slowly stain. The sections were then converted into digital photographs, and measurements of the myocardial infarction area (pale), ischemic risk area (red) and the non-infarct area (blue) carried out using the Image Pro plus 6.0 software.

### Experimental procedures

The diabetic rats were randomly divided into sham operation group (Sham group), myocardial ischemia–reperfusion group (I/R group), myocardial ischemia–reperfusion + Adenosine receptor A2 agonist group (I/R + NECA group), myocardial ischemia–reperfusion + NECA + ERS inducer group (I/R + NECA + TM group), myocardial ischemia–reperfusion + adenosine receptor A2 agonist + ERS inducer group + IRE1 inhibitor group (I/R + NECA + TM + STF group). NECA was administered 10 min before ischemia.

The cardiomyocytes HC9c2 after high glucose treatments were randomly divided into sham operation group (HC9c2 + high glucose), Hypoxia-reperfusion group (HC9c2 + high glucose + H/R), myocardial ischemia–reperfusion + Adenosine receptor A2 agonist group (HC9c2 + high glucose + NECA + H/R), myocardial ischemia–reperfusion + NECA + ERS inducer group (HC9c2 + high glucose + NECA + Tunicamycin + H/R), myocardial ischemia–reperfusion + adenosine receptor A2 agonist + ERS inducer group + IRE1 inhibitor group (HC9c2 + high glucose + NECA + Tunicamycin + STF + H/R). NECA was administered 10 min before hypoxia.

### Protein extraction and western blot

Radio immunoprecipitation assay (RIPA) lysis buffer (Beyotime Biotechnology, Shanghai, China), mixed with protease inhibitor, phenylmethylsulfonyl fluoride (PMSF) (Thermofischer scientific, USA) was used to extract total protein from cells and tissues. Final protein concentration was measured using Bicinchoninic acid (BCA) assay kit (Beyotime Biotechnology, Shanghai, China). Appropriate concentrations were determined and the proteins loaded onto 10% SDS PAGE gels and separated by electrophoresis. The proteins were then transferred on to polyvinylidene difluoride (PVDF) membranes, (Sigma-Aldrich, Beijing, China) incubated in primary antibodies overnight at 4 °C, then incubated in appropriate secondary antibodies for 1 h at room temperature and finally target bands visualized using the ECL imaging system (model 5200, Tianneng, China).

### Detection of cardiac troponin and creatine kinase-MB levels

Enzyme linked immunosorbent assay (ELISA) was used to detect indicators of cardiomyocyte injury, CK-MB (creatine kinase-MB) and cTnI (cardiac troponin I), using kits obtained from CUSABIO BIOTECH Co., Ltd., Wuhan, China following the corresponding manufacturer’s instruction. 4 ml of blood samples were tapped into EDTA anticoagulant tubes from the femoral vein 2 h post reperfusion, centrifuged at 2000 rpm for 20 min at 4 °C and ELISA conducted.

### Hematoxylin and Eosin staining

Heart tissues quickly harvested after reperfusion were washed 3 times in sterile PBS, and fixed using formalin for 24 h. The fixed tissues were then successively dehydrated in ethanol of increasing concentration; 70%, 80%, 90%, 95%, and 100%, then washed in xylene before paraffin embedding. 5 μm paraffin tissue blocks were prepared, and the tissue sections progressively dewaxed in xylene. They were then dehydrated in decreasing concentration of ethanol; 100%, 95%, 80%, and 75%), and then stained using hematoxylin and eosin. The tissues were then dehydrated, and sealed with neutral resins, and observed under the microscope.

### Tunel assay

To further demonstrate apoptosis, Tunnel assay was conducted. Briefly, cells were grown on small glass slides inside a six well plate. The cells were fixed with 4% formaldehyde, washed 3 times with PBS, incubated in 70% ethanol for 30 min at 4 °C, again washed again 3 times with PBS, then incubated in a mixture of TdT enzyme and dUTP for 60 min at 37 °C. The cells were then rinsed with PBS, and incubated with DAPI counter stain for 10 min at room temperature and kept in the dark. They were then washed 3 times in PBS and re incubated with 7-AAD / RNase A solution and incubated for 30 min at room temperature then observed under a fluorescence microscope and images collected.

### Electron microscopy

Following reoxygenation, cells were fixed in 2.5% glutaraldehyde for 4 h at 4 °C, rinsed 3 times using 0.1 M phosphoric acid buffer then fixed for 2 h in 1% osmic acid. Gradual dehydration in increasing concentration of ethanol was conducted; (50%, 70%, 90%, 100%), then permeated, and embedded in epoxy resin for 48 h at 60 °C. An ultrathin slicer (Leica, EM UC7, German) was used to make 80 nm slices of the embedded tissues and then stained with 2% uranyl acetate and lead citrate. Cellular morphology and autophagic flux were observed under an electron transmission microscope (Hitachi TEM system, HT7800, Japan).

### Statistical analysis

All data were expressed as mean ± standard error of the mean unless explicitly specified, and analyzed statistically using GraphPad Prism 8.0 (GraphPad Software, Inc., La Jolla, CA). Normality tests were conducted and data normalized to the sham/control group. The unpaired Student's t test was used to compare the differences between two groups, and one-way ANOVA used to verify differences among more than two groups with Bonferroni or Dunnett post-hoc conducted, unless otherwise stated. P value of < 0.05 was considered significant.

## Results

### Activation of A2R facilitated myocardial function recovery after MI/RI in diabetic rats

**Fig. 1:**
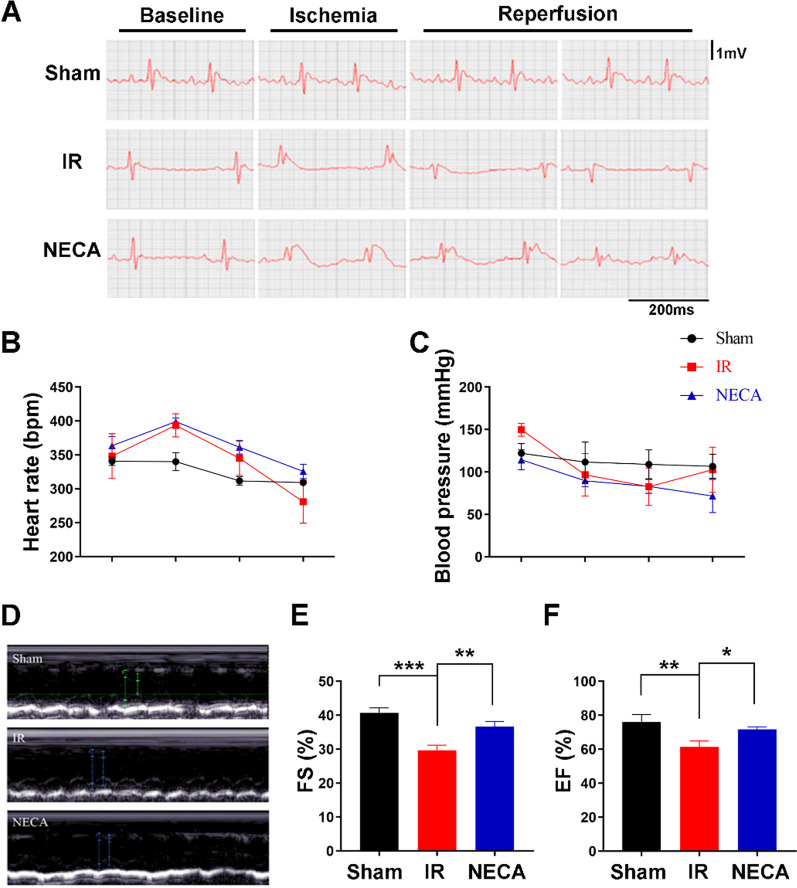
Dynamic electrocardiogram and hemodynamic monitoring of the heart. **A**ECG performance at baseline, ischemia, and reperfusion for each group. **B** Changes in heart rate at each time point expressed as (X ± SD); **C** Changes in mean arterial pressure in each group (X ± SD). **D** Echocardiographic manifestations of each group; **E**, **F** Left ventricular ejection fraction (LVEF) and left ventricular shortening fraction (FS) of rats in each group compared with control group (sham) and IR group (IR), ** P < 0.01

To evaluate the effect of adenosine A2a receptor activation on myocardial reperfusion injury in diabetic rats, an in vivo diabetic rat model of MIRI was established, and NECA, a potent, non-selective A2R agonist was administered to activate A2R prior to reperfusion. Successful establishment of the model was confirmed by using an electrocardiogram (ECG), as indicated by the presence of ST segment elevation on the ECG following left anterior descending coronary artery (LAD) ligation of the rats. After 120 min of reperfusion, there was an obvious inverted Q wave and a prolonged QT interval in the ECG of the I/R group of rats. In contrast, QT interval prolongation was relatively improved in the I/R + NECA group (Fig. 1A). The changes in heart rate and mean arterial pressure during I/R were not statistically significant among the groups, (Fig. 1B, C). These results suggested that activation of adenosine A2a receptor prior to reperfusion, facilitated quick ventricular repolarization after ischemia of the myocardium.

About five minutes to the end of reperfusion, cardiac ultrasound was conducted to observe changes in cardiac functions of the rats, (Fig. 1D). Echocardiography revealed that the left ventricular ejection function (LVEF) and shortening fraction (lvfs) in I/R group were significantly lower than those in the sham group (Fig. 1E, F; P < 0.05), indicating that I/R significantly damaged the systolic function of the heart. Compared with IR group, LVEF and lvfs in I/R + NECA group were significantly improved (Fig. 1E, F; P < 0.05). This indicates that the activation of adenosine A2a receptor significantly facilitated recovery of cardiac systolic function.

### Activation of adenosine A2R attenuated MIRI in diabetic rats

**Fig. 2:**
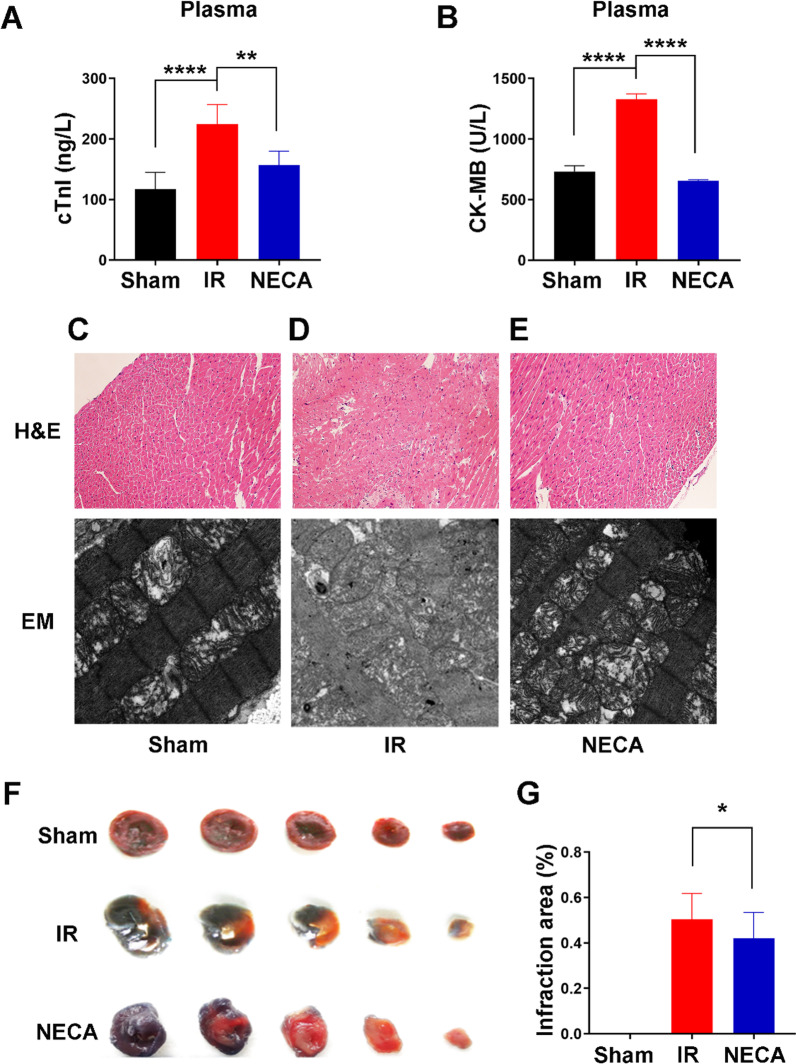
Adenosine A2 receptors activation attenuated MI/RI in the rats. **A**, **B** Plasma levels of cTnI and CK-MB in the different groups of rats. Data were presented as mean ± SD and analyzed by one-way ANOVA with uncorrected Fisher’s LSD post hoc test, ***P < 0.001, and ****P < 0.0001 respectively. **C–E** H&E staining and electron microscopy of the cardiac tissues. **F**, **G** Comparative percentage of myocardial ischemic area in each group. Infarct size was significantly reduced in the IR + NECA group versus IR group, * P < 0.05

To determine the effects of A2R activation on myocardial damage following ischemia, Serum levels of the myocardial enzymes creatinine kinase MB (CK-MB) and cardiac troponin (cTnI) were determined. CK-MB was significantly increased following ischemia, from 731.45 ± 49.61 in the sham group to 1329.30 ± 44.23 in the IR group. This was dramatically reduced to 656.91 ± 7.02 in I/R + NECA group. Similarly, cTnI increased following ischemia from 117.26 ± 27.53 in the sham group to 215.98 ± 42.97 in I/R group. This was also significantly reduced to 165.55 ± 30.36 in I/R + NECA group, demonstrating that A2R activation by NECA attenuated ischemic myocardial damage (Fig. 2A, B). Hematoxylin and eosin (H&E) staining and electron microcopy were then conducted on the myocardial tissues obtained before and after reperfusion. The results revealed that the myocardial damage seen in the I/R group was almost completely reversed after A2R activation prior to reperfusion in the I/R + NECA group (Fig. 2C–E). To determine the size of myocardial infarction area, Evans Blue staining was used. Compared to the IR group, the myocardial infarction area in the I/R + NECA group decreased by about 41% (Fig. 2F, G). The ischemic area of the two groups did not differ significantly signifying consistency in the LAD ligation sites of the models. Taken together, these result demonstrated that activation of adenosine A2a receptor using NECA prior to reperfusion attenuated myocardial ischemia reperfusion injury in the diabetic rats.

### Activation of A2R protected the myocardium through regulating endoplasmic reticulum stress-induced autophagy and apoptosis

**Fig. 3:**
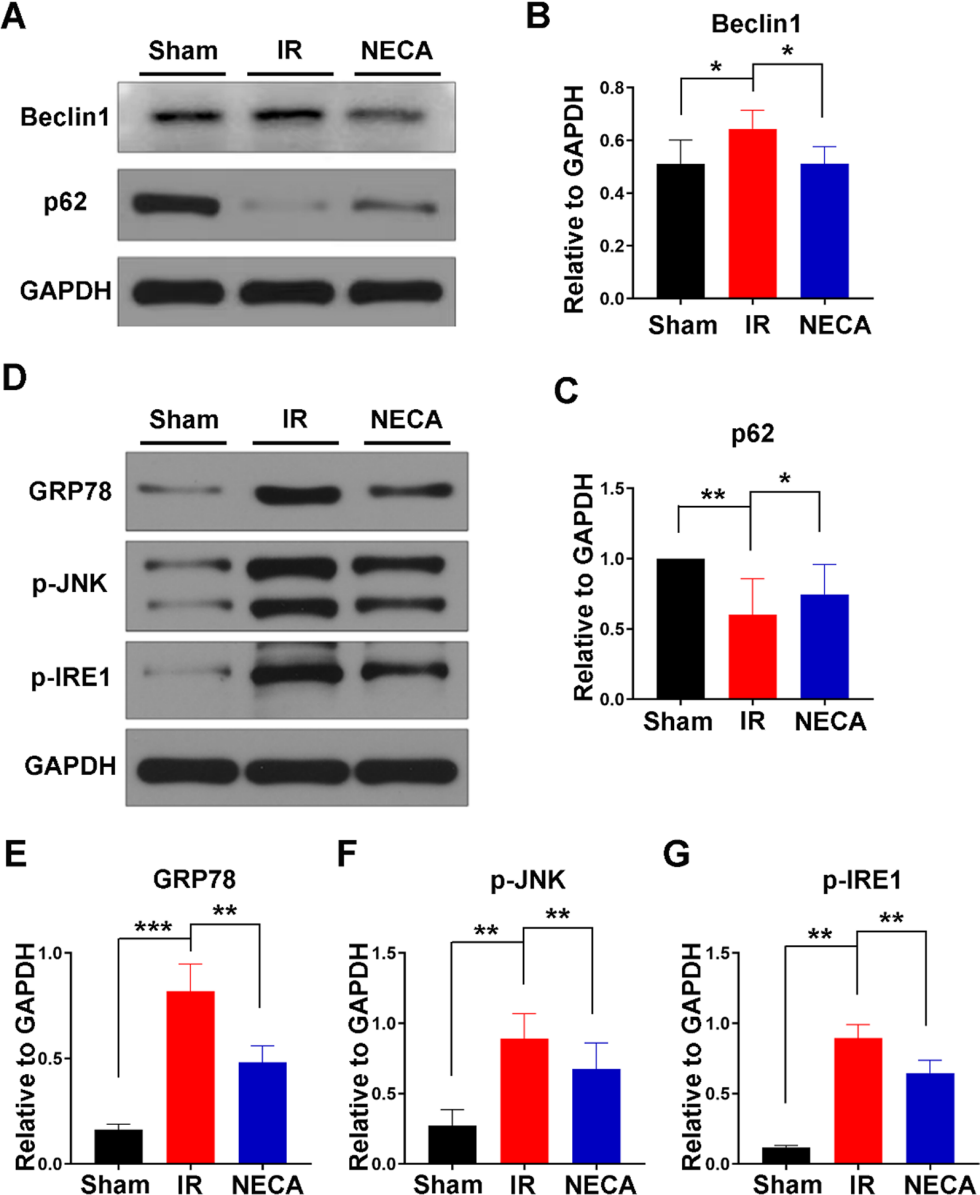
Effects of A2R activation on autophagy and ERS. **A–C** Expression of autophagy proteins Beclin and p62. Beclin is decreased while p62 decline is attenuated by A2R activation in the IR + NECA group compared to sham group and the IR group, * P < 0.05, ** P < 0.01. **D–G** ERS proteins (GRP78, p-IRE-1/IRE1, p-JNK / JNK) are increased in IR group but administration of NECA significantly reduced their expression in the IR + NECA group, **P < 0.01

To explore the roles of the endoplasmic reticulum stress (ERS) induced autophagy in MIRI in the diabetic rats in response to A2R activation, its agonist NECA was administered 1 h prior to reoxygenation. NECA administration in the I/R + NECA group significantly decreased the expression of the autophagy promoting protein, Beclin-1 compared to the I/R and the sham groups. On the other hand, its administration attenuated the decrease in the autophagosome substrate p62. (Fig. 3A–C). These results indicated that A2R activation had an anti-autophagy effect following ERS induction. ERS was determined by measuring the expression levels of ERS related genes GRP78, p-IRE1/IRE1, and p-JNK/JNK and their corresponding protein levels. Increase in GRP78, p-IRE1/IRE1, and p-JNK/JNK in the IR group of rats indicated increase in ERS induced apoptosis via the IRE-1/JNK pathway, which was significantly attenuated by A2R activation in the IR + NECA group (Fig. 3D–G). The above results demonstrated that A2R activation could have protected the myocardium during MIRI through regulating ERS induced autophagy.

**Fig. 4:**
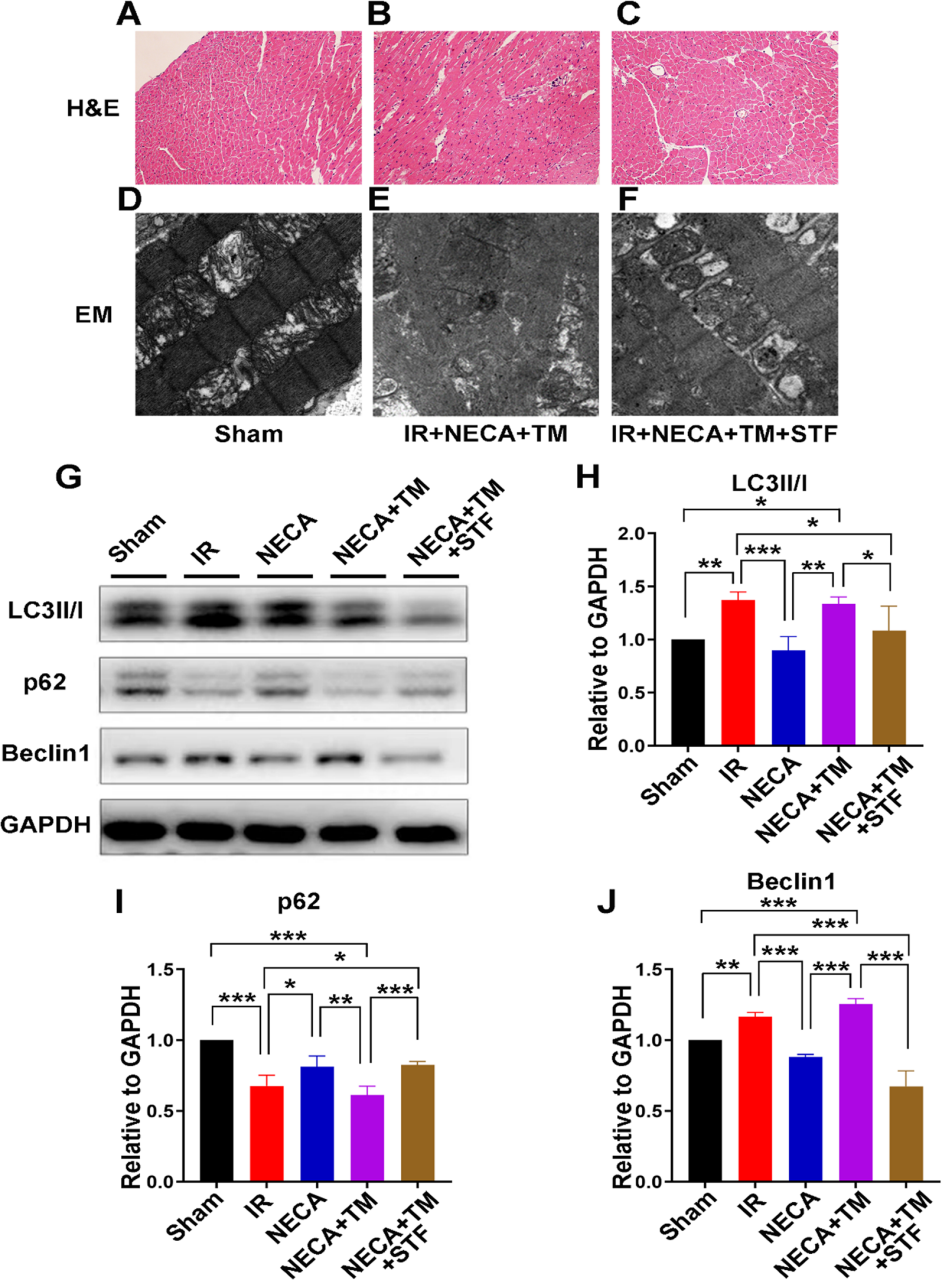
H&E staining, **A** In the Sham group, the myocardial fibers are complete; the Z-line is clear, continuous and straight, and the structural position is normal. **B** After ERS induction with TM, the heart muscle fibers were broken, Z-line was interrupted and discontinuous, and the mitochondria shifted and vacuolation increased. **C** After administration of IRE-1 inhibitor before reperfusion, the myocardial fibers were again continuous, Z-line was continuous, and mitochondrial edema and vacuolation were reduced. Electron microscopy: **D** In the Sham group, the myocardial fibers are complete; the mitochondria normal, and the cristae are clear. **E** After ERS induction using TM, muscle fibers were broken, Z-line was interrupted and discontinuous, mitochondria shifted and vacuolation increased; **F** After administration of IRE-1 inhibitor prior to reperfusion, the, myocardial fibers were continuous again, Z-line was continuous, and mitochondrial edema and vacuolation were reduced. **G**–**J** Administration of IRE-1 inhibitor significantly reduced the expression levels of Beclin-1, prevented the conversion of LC3I to II and attenuated the reduction of p62

To explore the mechanism by which A2R activation influences the endoplasmic reticulum stress induced autophagy to confer cardio protection in MIRI, the ER resident protein IRE-1 was modulated. Previous studies have shown that IRE-1 regulates ER-stress induced autophagy in various cells [13–15]. The ER-stress inducer Tunicamycin (TM), and the IRE-1 inhibitor STF in combination with the A2R agonist NECA, were used, and the cellular responses measured using expression of the autophagy proteins Beclin-1, p62 and LC3. H&E staining of the I/R + NECA + TM group revealed broken muscle fibers, interrupted and discontinuous Z-line, and shifted & vacuolated mitochondria indicating induction of ER stress, (Fig. 4A–C). These were however all reversed following administration of the IRE-1 inhibitor in the I/R + NECA + TM + STF group; the myocardial fibers were continuous, Z-line was continuous, and mitochondrial edema and vacuolation were significantly reduced. Similar results were observed with the electron microscopy, indicating that inhibition of IRE-1 alleviated ER-stress, (Fig. 4D–F). In the western blot analysis, the administration of IRE-1 inhibitor prior to induction of ERS by TM, significantly reduced the expression levels of Beclin-1, prevented the conversion of LC3I to II and attenuated the reduction of p62, indicating attenuation of ERS-induced autophagy (Fig. 4G–J). Moreover, the protein expression levels of the IRE1 signaling pathway-related proteins GRP78, IRE1, and p-JNK were substantially raised during I/R injury yet significantly decreased following treatment with STF These results indicated that inhibition of IRE-1attenuated ERS-induced autophagy in the diabetic rats (Fig. 3D–G).

### A2R activation attenuated ERS induced apoptosis under hypoxia/reperfusion

**Fig. 5:**
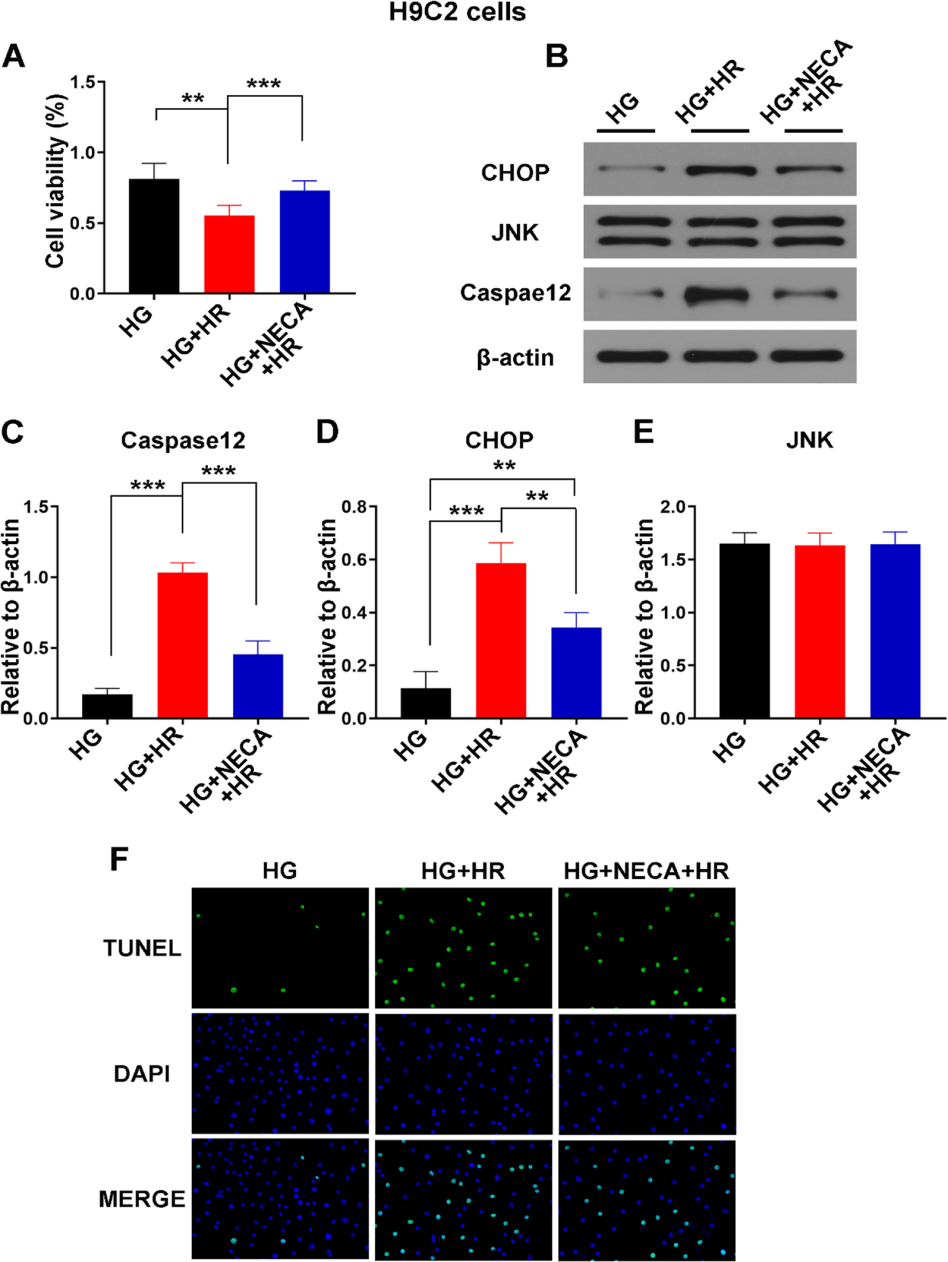
Effect of A2R activation on autophagy. **A** Cell viability was detected by CCK-8. **B**–**E** Western blot analysis of apoptosis related proteins Caspase-12, CHOP, and JNK analyzed by Image J. All data presented as mean ± SEM and analyzed by one-way ANOVA and Dunnett’s post hoc test; n = 3. **P < 0.01, ***P < 0.001 and *P < 0.05 respectively. **F** TUNEL staining demonstrating apoptosis

To further assess the regulatory effects of adenosine A2 receptor agonists on endoplasmic reticulum stress induced autophagy and apoptosis in MIRI and the interaction between the two mechanisms, an in vitro ischemia reperfusion injury model in diabetic cardiomyocytes was developed. H9C2 cardiomyocytes cell line was used. Exposing the H9C2 cells to hypoxia induced significant cell death, which was alleviated by administration of the adenosine A2R agonist NECA (Fig. 5A). Apoptosis related proteins CHOP, and Caspase 12 were significantly increased in the H9C2 + high glucose + H/R group compared to Sham group, and sharply decreased following administration of the adenosine A2R agonist, NECA in the H9C2 + high glucose + H/R + NECA group (Fig. 5B–D). However, the total amount of JNK did not change (Fig. 5B, E), while its activated form that is known to induce apoptosis [16], significantly increased in the I/R group following reperfusion and was attenuated by A2R activation in the IR + NECA group (Fig. 3D, F). Similarly, tunnel staining indicated increased apoptosis in the H9C2 + high glucose + H/R group, which was alleviated in the H9C2 + Glu + high glucose + NECA group (Fig. 5F).

### Cardio-protection effect of A2R activation in MIRI is by regulating endoplasmic reticulum stress induced autophagy through IRE-1 protein

**Fig. 6:**
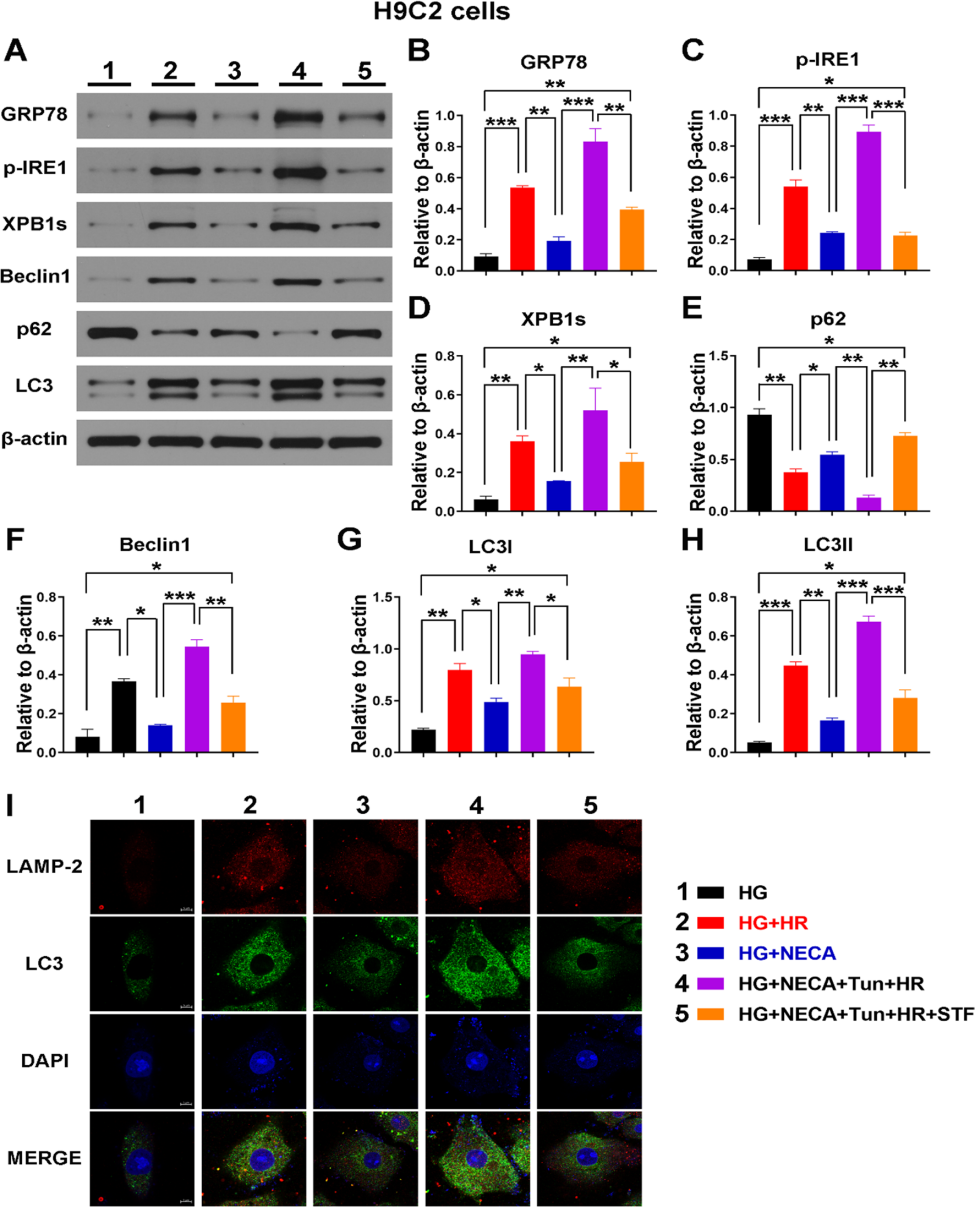
Effect of A2R activation on autophagy and endoplasmic reticulum stress. **A**–**D** Expression levels ERS proteins GRP78, p-IRE-1 and XBP1s evaluated by western blot and analyzed by image J. *P < 0.05, **P < 0.01, ***P < 0.001. **E**–**H** Expression levels of autophagy proteins, p62, Beclin-1, LC3I and LC3 II evaluated by western blot and analyzed by image J. ***P < 0.001, ****P < 0.0001. All data presented as mean ± SEM, analyzed by one way ANOVA and Tukey’s post hoc test. **I** LC3-LAMP-2 co-localization viewed by fluorescence microscopy

To investigate the protective role of A2R in H/R, its effect on endoplasmic reticulum stress, and autophagy was evaluated. NECA was used to activate A2R while Tunicamycin was used to induce endoplasmic reticulum stress (ERS) and the levels of ERS related proteins GRP78, p-ire1and XBP1s determined. In addition, p-IRE-1 inhibitor STF was used to evaluate the possible involvement of p-IRE-1 protein in regulating autophagy in response to ERS. As expected, the levels of ERS proteins GRP78, p-IRE1and XBP1 were all increased in response to Tunicamycin treatment (Fig. 6A–D), while the levels of autophagy related proteins Beclin-1 and LC3 were increased and the autophagosome substrate protein, p62 decreased indicating increased autophagy (Fig. 6E–H). Similarly, increased co-localization of LC3 and LAMP-2 was observed in the ERS induced group using immunofluorescence staining (Fig. 6I) indicating that ERS induction promoted autophagosomes and lysosomes fusion. These were however all reversed following treatment with p-IRE-1 inhibitor STF, indicating that p-IRE-1 could be involved in the regulation of ERS induced autophagy.

## Discussion

MIRI remains a major cause of death of cardiomyocytes in patients with coronary heart disease [17]. Mechanisms responsible for cardiomyocytes death during MIRI include; autophagy, apoptosis, ferroptosis and pyroptosis [18]. Autophagy is a highly conserved cellular process where nutrients are catabolized and recycled through lysosomal degradation of unfolded or misfolded proteins and worn out cellular organelles [11]. It releases amino acids, fatty acids, ATP, and other high energy molecules that are essential for cell survival and normal tissue function during ischemia or nutrient deficiency [11]. This is particularly important for non-proliferative cells such as cardiomyocytes as it ensures cell survival and maintains structural and functional stability of the heart. The endoplasmic reticulum (ER) is one of the major cellular organelles that regulates autophagy [19]. Under extreme external stress, protein folding in the ER is disrupted and so misfolded and unfolded proteins accumulate in the ER lumen and the cytoplasm. These proteins interact with p62, which then recruits LC3II [20, 21], and together form autophagosomes. In the presence of LAMP2, these autophagosomes fuse with lysosomes to form autolysosomes leading to lysis and recycling of the contents [22, 23], and eventual survival of the cell. This process is called endoplasmic reticulum stress (ERS) induced autophagy. However, autophagy is a double edged sword; enhanced autophagy following MIRI can lead to significant reduction of viable cardiomyocytes which impairs recovery of ventricular functions and exacerbate the condition [24–27].

Given the upregulation of CHOP and caspase 12 in our experiment, we investigated ERS involvement in the resultant autophagy and apoptosis using the ERS inducer Tunicamycin. Induction of ERS resulted into increased expression of BiP (GRP78), p-IRE1 and XBP1s. The response of CHOP to ERS is often regulated by three factors; protein kinase RNA-like endoplasmic reticulum kinase (PERK), activating transcription factor 6 (ATF6), and inositol requiring protein 1 (IRE1) [28]. The dramatic increase in the expression of IRE1 here suggested that it was the likely pathway in this experiment. IRE1 is kept in its inactive state by GRP78 interaction. Upon accumulation of unfolded proteins, GRP78, having more affinity for unfolded proteins, disintegrates from IRE1 thus activating it [29]. In its active form, IRE1 cleaves its substrate precursor XBP1 to its active form XBP1s [30, 31]. XBP1s then upregulates the expression of CHOP and many other genes involved in UPR to restore protein homeostasis [32–34]. However, when ERS is prolonged or severe, IRE1 stimulates the activation of the apoptotic-signaling kinase-1 (ASK1), which in turn activates JNK and p38 MAK hence inducing apoptosis [35]. To confirm the involvement of IRE1, the cardiomyocytes were treated with the IRE1 inhibitor STF. This significantly inhibited the autophagy promoter Beclin-1 and resulted into accumulation of p62 indicating reduced or inhibited autophagy.

In this experiment, subjecting the cardiomyocytes to hypoxia and reoxygenation (H/R) induced significant apoptosis as demonstrated by the CCK-8 assay and the dramatic increase in apoptosis related proteins CHOP and caspase-12. CHOP and caspase-12 are particularly associated with endoplasmic reticulum stress (ERS) induced apoptosis [36, 37]. Since JNK levels remained constant, this apoptosis was most likely independent of the JNK pathway. ERS-induced apoptosis follows three major pathway; the IRE1/ASK1/JNK pathway, the caspase-12 kinase pathway, and the C/EBP homologous protein (CHOP)/GADD153 pathway [28, 30], so it was likely that the later pathway was involved. Activation of A2R using its agonist NECA swiftly reversed apoptosis. This suggested that NECA could be a very useful therapeutic for early stage MIRI management in diabetics. The finding are consistent with our previous results [9, 38] which demonstrated that A2aR activation protects the myocardium against MIRI through downregulating autophagy and regulating autophagy flux and apoptosis in non-diabetic cardiomyocytes. In our most recent work, [10], we demonstrated that NECA attenuated MIRI in type-2 diabetic rats through A2aR/PCK/miR-15a mechanism. However, we speculated that the autophagic flux seen by Xia et al. [38] in nondiabetic rats could also occur in type-2 diabetic rats, and decided to use NECA, a non-selective activator of adenosine receptors to demonstrate ERS involvement in this cardio protection. This results has thus demonstrated that indeed A2R activation in type-2 diabetic rats by NECA could also be cardioprotective via ERS inhibition mechanisms.

In the animal experiment, myocardial ischemia (MI) resulted into leakage of cTnI and CK-MB into the blood stream and infarction of the myocardium. These myocardial damages were promptly reversed by activation of A2R by NECA. Similarly, the ventricular systolic function that was impaired by MI also resolved following A2R activation. MI also impaired the LVEF and LVFS indicating impaired ventricular systolic function. However, A2R activation ameliorated both conditions, in effect restoring left ventricular systolic function. Studies have shown that recovery of ventricular systolic function following MIRI is dependent on A2R inducing the elevation of cAMP and PKA, which then triggers multiple cAMP-PKA dependent calcium ion channels, heightening the maximum peak transient outward current thus enabling post ischemia repolarization of the ventricles [39–41]. Therefore, cAMP mediated influx of Ca2 + promotes reestablishment of myocardial contractility.

On the electrocardiogram, MI elevated the ST segment and caused prolongation of the QT interval. This was possibly caused by the imbalance in the in and out flows of intracellular and extracellular ion (Ca2 + , K + , H + , and Na +), due to the interrupted blood and oxygen supply following ischemia, hence changing the repolarization current (42). This current change in the ischemic area thus prolonged the QT wave and elevated the ST-segment. Activation of A2R during ischemic post conditioning has an antiarrhythmic effect associated with action potential shortening [44, 45].

This study had the following limitations: (1) we have previously demonstrated that A2aR activation protects the myocardium against MIRI through regulating autophagy flux and apoptosis in non-diabetic cardiomyocytes, and that NECA attenuates MIRI in type-2 diabetic rats through A2aR/PCK/miR-15a mechanism [10, 38]. Here, to investigate ERS involvement in MIRI in diabetic rats we used NECA, a nonselective A2R activator. Our result here is therefore, not specific to which adenosine receptor subtype is responsible for this ERS involved cardio protection in type-2 diabetic rats. In a future study, this will be resolved. (2) According to the IMproving Preclinical Assessment of Cardioprotective Therapies (IMPACT) criteria by the European Union-CARDIOPROTECTION COST Action [46], in small animal models of acute ischemia reperfusion injury (IRI), it is desirable to demonstrate benefit of the intervention after at least 28 days post intervention. However, this was not the case in this study, therefore these results should be interpreted accordingly.

## Conclusions

In summary, activation of A2R prior to reperfusion following myocardial ischemia effectively attenuated apoptosis, reduced ERS induced autophagy and re-established impaired left ventricular function, hence reducing the damaging effects of MIRI on diabetic cardiomyocytes. This protective effect occurs through regulating the IRE1-XBPs-CHOP related mechanisms.

## Supplementary Information

**Additional file 1**.** Table S1**. Information on all the primers used for qPCR in the study.

**Additional file 2**.** Figure F1**. Original blots for the effects of A2R activation on autophagy and ERS. The autophagy proteins Beclin 1 and P62 were assessed. The 60Kda protein is Beclin 1, 62Kda protein is P62 and the 36Kda protein is GAPDH; the internal control protein.

**Additional file 3**.** Figure F2**. Original blots for the effects of A2R activation on autophagy and ERS. The ERS proteins Grp78, P-JKN, and P-IRE1 were assessed. The 78Kda protein is Grp78, 54Kda and 46Kda proteins are isoforms of P-JKN, and the 110Kda protein is P-IRE1. The 36Kda protein is GAPDH; the internal control protein.

**Additional file 4**.** Figure F3**. Original blots of autophagy proteins measured in response to P-IRE1 inhibition. The 16Kda protein is LC3, the 62Kda protein is P62 and the 60Kda protein is Beclin 1. The 36Kda protein is GAPDH as the internal control protein.

**Additional file 5**.** Figure F4**. Original blots of apoptosis related proteins in H9C2 cells following hypoxia. The proteins assessed were CHOP, JKN, and Caspase 12. The 27Kda protein is CHOP, the 54Kda and 46Kda proteins are isoforms of JKN and the 46Kda protein is Caspase 12. The 43Kda protein is Beta-actin; the internal control protein.

**Additional file 6**.** Figure F5**. Original blots of autophagy and ERS proteins in H9C2 cells following activation of A2R. The autophagy proteins assessed were Beclin 1, p62, and LC3, while the ERS proteins were Grp78, P-IRE1 and XBP1s. Beta actin was the internal control protein.

## Data Availability

All the supporting data are available with the corresponding author and shall be made available upon request.
