# Supplementary material for: Adenosine 2 receptor regulates autophagy and apoptosis to alleviate ischemia reperfusion injury in type 2 diabetes via IRE-1 signaling
Source: BMC Cardiovasc Disord. 2023 Mar 24;23:154. doi: 10.1186/s12872-023-03116-y (PMC10039586; doi:10.1186/s12872-023-03116-y)
Supplement: Supplementary file 1 — Additional file 1. Table S1. Information on all the primers used for qPCR in the study. [file 12872_2023_3116_MOESM1_ESM.docx]

**Additional file 1**

**Table S1. PCR primers**

| **Gene Name** | **Primer information** | **Sequence（5‘-3’）** | | **Tm value** | **CG%** |  |
| --- | --- | --- | --- | --- | --- | --- |
| B-ACTIN | NM_031144.3 | Sense | CGTTGACATCCGTAAAGACCTC | 59 | 50 | 110 |
|  |  | Antisense | TAGGAGCCAGGGCAGTAATCT | 58.4 | 52.4 |  |
| R-A2A | NM_171983 | Sense | CTATGCCAAGGAAGAAGTAGCAG | 58.9 | 47.8 | 213 |
|  |  | Antisense | TGGACATTAGTGAGGTCTTTACCAT | 59.5 | 40 |  |
| R-A2BR | NM_017161 | Sense | TGCTCACACAGAGCTCCATCTT | 61 | 50 | 246 |
|  |  | Antisense | TCTCAAAGAGACACTTCACAGGG | 60 | 47.8 |  |
