# Supplementary figures and images for: Adenosine 2 receptor regulates autophagy and apoptosis to alleviate ischemia reperfusion injury in type 2 diabetes via IRE-1 signaling
Source: BMC Cardiovasc Disord. 2023 Mar 24;23:154. doi: 10.1186/s12872-023-03116-y (PMC10039586; doi:10.1186/s12872-023-03116-y)

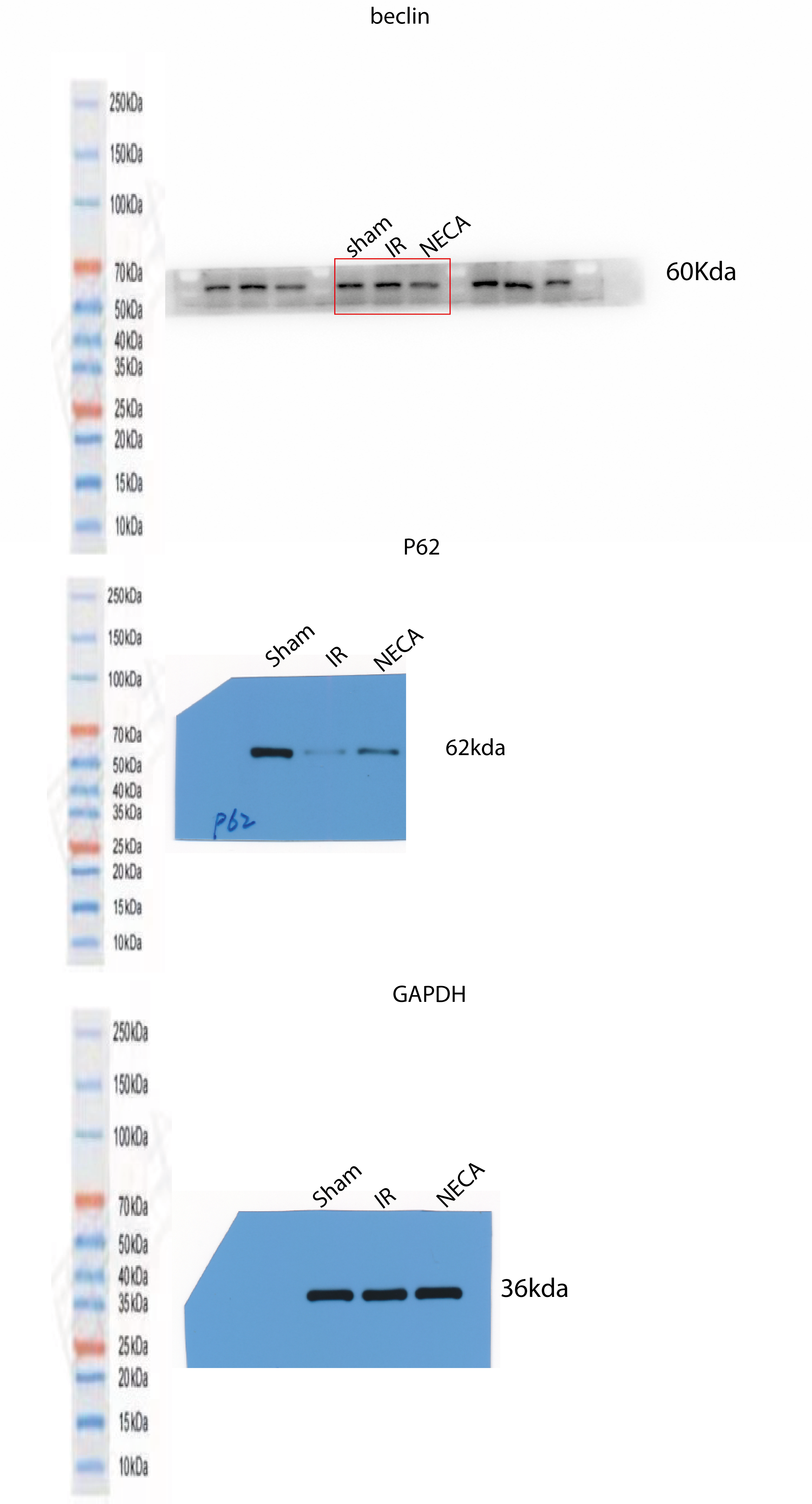

Supplement: Supplementary file 2 — Additional file 2. Figure F1. Original blots for the effects of A2R activation on autophagy and ERS. The autophagy proteins Beclin 1 and P62 were assessed. The 60Kda protein is Beclin 1, 62Kda protein is P62 and the 36Kda protein is GAPDH; the internal control protein. [file 12872_2023_3116_MOESM2_ESM.tif]

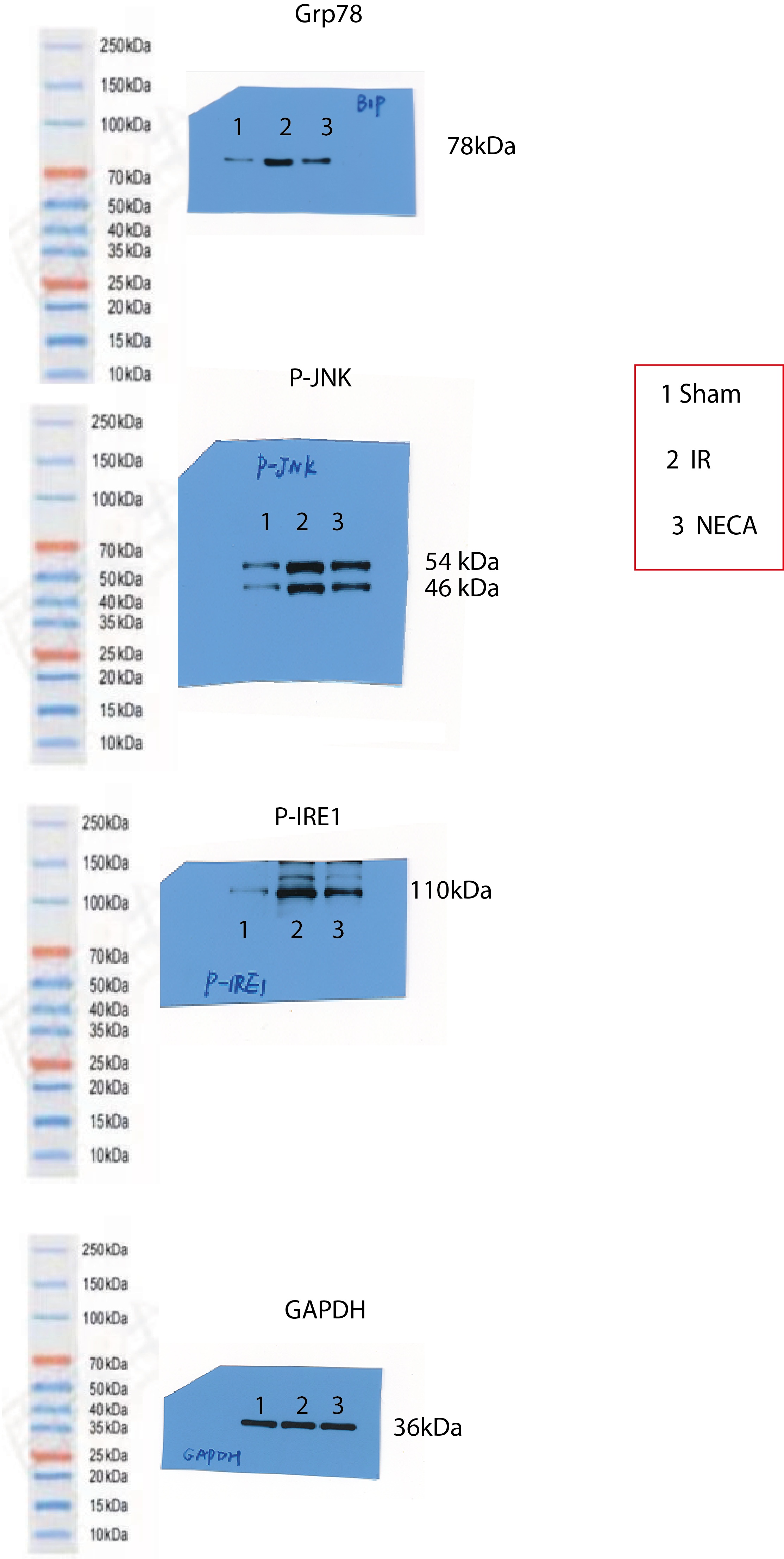

Supplement: Supplementary file 3 — Additional file 3. Figure F2. Original blots for the effects of A2R activation on autophagy and ERS. The ERS proteins Grp78, P-JKN, and P-IRE1 were assessed. The 78Kda protein is Grp78, 54Kda and 46Kda proteins are isoforms of P-JKN, and the 110Kda protein is P-IRE1. The 36Kda protein is GAPDH; the internal control protein. [file 12872_2023_3116_MOESM3_ESM.tif]

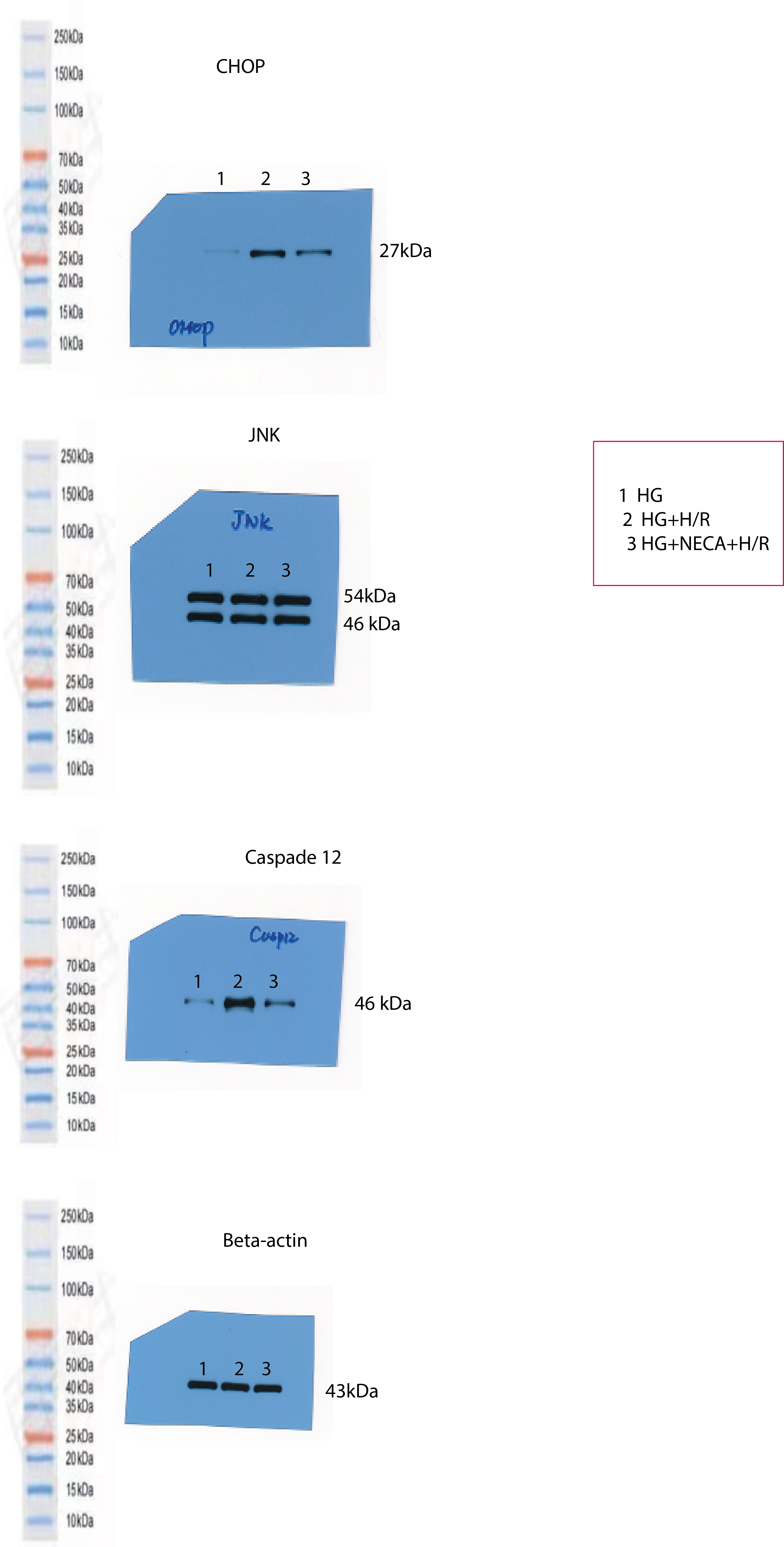

Supplement: Supplementary file 5 — Additional file 5. Figure F4. Original blots of apoptosis related proteins in H9C2 cells following hypoxia. The proteins assessed were CHOP, JKN, and Caspase 12. The 27Kda protein is CHOP, the 54Kda and 46Kda proteins are isoforms of JKN and the 46Kda protein is Caspase 12. The 43Kda protein is Beta-actin; the internal control protein. [file 12872_2023_3116_MOESM5_ESM.tif]
